# Evolutionary Dynamics on Protein Bi-stability Landscapes can Potentially Resolve Adaptive Conflicts

**DOI:** 10.1371/journal.pcbi.1002659

**Published:** 2012-09-13

**Authors:** Tobias Sikosek, Erich Bornberg-Bauer, Hue Sun Chan

**Affiliations:** 1 Evolutionary Bioinformatics Group, Institute for Evolution and Biodiversity, University of Münster, Münster, Germany; 2 Departments of Biochemistry, Molecular Genetics, and Physics, University of Toronto, Toronto, Ontario, Canada; University of Texas at Austin, United States of America

## Abstract

Experimental studies have shown that some proteins exist in two alternative native-state conformations. It has been proposed that such bi-stable proteins can potentially function as evolutionary bridges at the interface between two neutral networks of protein sequences that fold uniquely into the two different native conformations. Under adaptive conflict scenarios, bi-stable proteins may be of particular advantage if they simultaneously provide two beneficial biological functions. However, computational models that simulate protein structure evolution do not yet recognize the importance of bi-stability. Here we use a biophysical model to analyze sequence space to identify bi-stable or multi-stable proteins with two or more equally stable native-state structures. The inclusion of such proteins enhances phenotype connectivity between neutral networks in sequence space. Consideration of the sequence space neighborhood of bridge proteins revealed that bi-stability decreases gradually with each mutation that takes the sequence further away from an exactly bi-stable protein. With relaxed selection pressures, we found that bi-stable proteins in our model are highly successful under simulated adaptive conflict. Inspired by these model predictions, we developed a method to identify real proteins in the PDB with bridge-like properties, and have verified a clear bi-stability gradient for a series of mutants studied by Alexander et al. (Proc Nat Acad Sci USA 2009, 106:21149–21154) that connect two sequences that fold uniquely into two different native structures via a bridge-like intermediate mutant sequence. Based on these findings, new testable predictions for future studies on protein bi-stability and evolution are discussed.

## Author Summary

Proteins are essential molecules for performing a majority of functions in all biological systems. These functions often depend on the three-dimensional structures of proteins. Here, we investigate a fundamental question in molecular evolution: how can proteins acquire new advantageous structures via mutations while not sacrificing their existing structures that are still needed? Some authors have suggested that the same protein may adopt two or more alternative structures, switch between them and thus perform different functions with each of the alternative structures. Intuitively, such a protein could provide an evolutionary compromise between conflicting demands for existing and new protein structures. Yet no theoretical study has systematically tackled the biophysical basis of such compromises during evolutionary processes. Here we devise a model of evolution that specifically recognizes protein molecules that can exist in several different stable structures. Our model demonstrates that proteins can indeed utilize multiple structures to satisfy conflicting evolutionary requirements. In light of these results, we identify data from known protein structures that are consistent with our predictions and suggest novel directions for future investigation.

## Introduction

New functional proteins are likely to evolve from existing proteins. Most existing proteins, however, are under selection to conserve their existing native structure in order to maintain functionality (and also to avoid aggregation and proteolysis). Without such selective constraints, the accumulation of random mutations would soon render a protein nonfunctional. When the same gene (protein) is under two selection pressures, i.e. to evolve a new functional structure while conserving its existing structure, an adaptive conflict arises. This adaptive conflict scenario is at the heart of most contemporary theories of molecular evolution, such as the popular Neofunctionalization and Subfunctionalization models (as reviewed in [1], [2]). However, these models generally require gene duplications to take place before adaptive conflicts can be resolved. This implies that such models can only explain long-term processes that involve many unlikely events, such as the occurrence of a suitable gene duplication event, followed by retention, fixation in the population, and additional beneficial or neutral point mutations in one or both gene copies. Only then would an adaptive advantage become possible. Because of these potential drawbacks, a more recent model (*Escape from Adaptive Conflict*, EAC) emphasizes the sufficiency of single-gene, multi-functional proteins during short term adaptive conflicts [3]. Similar ideas have been proposed earlier in terms of the concept of “gene sharing” [4], [5]. In fact, a gene duplication of a multi-functional protein is more likely to be successful than duplicating a protein with only a single function: first, because a new function is already present – thus it does not have to first evolve the new function in a rare mutant carrying a gene duplication; second, functional divergence can be faster because the multiple functions have already been responding to conflicting selection pressures; and, finally, retention and fixation of the duplication is more likely because the second copy can immediately provide higher activity levels through higher protein concentrations for the multiple protein functions, none of which would likely have been fully optimized in a single-gene, multi-functional protein.

Indeed, there is increasing evidence that proteins have a significant capacity for multi-functionality. Not only are many enzymes known to exhibit promiscuity for nonnative reactions and substrates [6]–[8], multi-functionality has also been linked to proteins with two or more stable conformations [9]–[12]. These proteins can be called bi- or multi-stable. A few naturally occurring cases of such proteins are known, such as the prion protein that can assume different structures. One of these structures can aggregate to cause neurodegenerative pathologies such as mad-cow and Creutzfeldt-Jakob diseases [13], [14]. Protein bi-stability was also found in the cysteine-rich domain proteins (minicollagen) that form the walls of Cnidarian organelles called nematocysts [15]. Different conformers of these protein domains exhibit distinct patterns of disulfide bridges and perform different functions. Another example is the antiviral protein RhTC, which was found to target different HIV viruses by allowing a dynamic active site to assume very different conformations [16]. More generally, emerging evidence is lending support to the view that functional promiscuity in enzymes may frequently be based on thermodynamic fluctuations of conformational sub-states [17]. However, this may not always be the case, for example, if the functional promiscuity is mediated by changes of catalytic residues that do not cause conformational changes. An evolutionary theory of structure-based multi-functionality requires detailed knowledge of the sequence-structure relationship in proteins, as emphasized by the theory of neutral networks [18]–[23]. A neutral network consists of a connected set of sequences that fold into the same native (maximally stable) structure, and a pair of sequences in the network is connected if and only if they differ by one point mutation. Proteins can tolerate a number of mutations (mostly of surface amino acids [24], [25]) without losing their native structure. It has been shown experimentally that the neutral networks of two protein structures can be directly connected, such that one or two mutations can cause a switch from one native structure to the other [9], [26]–[28].

Because actual protein sequence space is too vast for computational — let alone experimental — exploration using current resources, we rely on a well-established explicit-chain biophysical model with exhaustive sequence-to-structure mapping [22], [23], [29]–[33] to provide a model of protein sequence space consisting of sequences with up to six-fold degenerate native state (i.e. proteins with up to six native structures). This model, termed the “hydrophobic-polar” (HP) model, is based on the central role of hydrophobic interactions in protein structures [29]. Earlier studies using the HP model but with non-degenerate native states have revealed that sequence space consists of distinct islands of neutral networks corresponding to unique native structures, which can be bridged either by single-site mutations (substitutions) [22], [32] or recombinational jumps [30]. A key feature of neutral networks arising from the HP model and similar simple exact models is a funnel-like distribution of free energy values around a most stable, and mutationally robust, *prototype* sequence [23], [34]–[36], or consensus sequence [37]. These funnels can act as attractors on evolving proteins outside the neutral network by allowing for selection of excited (non-native) conformational states, the stabilities of which increase with every incremental step toward the prototype sequence of that excited state [32]. More recently, the model was used to show an association between evolvability and phenotypic variation [33]. Some sequences in HP and HP-like models have been shown to have multiple native structure [23], [29] and even exhibit prion-like behaviors [38], [39]. However, an extensive account of sequence spaces with degenerate native structures is lacking and most theoretical studies of protein neutral networks to date have not considered the implications of multiple native structures [40]–[47].

In this context, our main aims here are to investigate: (i) where do bridge proteins preferably locate in sequence space, (ii) the manner in which bi-stability is distributed in the sequence-space neighborhood around bridge proteins, and (iii) the role of opposing selection pressures in the evolutionary dynamics that may take advantage of bi-stability. Toward these goals, we will first describe below the characteristics of the sequence space in our simple biophysical protein chain model. We will show that bridge proteins, and bi-stable proteins in general, have a high potential for facilitating evolution under adaptive conflicts. We will further demonstrate that this potential originates from a nonrandom distribution of bi-stability in sequence space. Subsequently, we will apply the concepts and insights gained from our simple model to real protein structures. In particular, we will describe bi-stability in a well-documented experimental case and also in a larger set of putative bi-stable proteins in the Protein Data Bank (PDB).

## Results

### Sequence space distribution of bridge proteins

#### Proteins with degenerate native states as bridges between neutral networks

*Bridge proteins* have been described as intermediate evolutionary states along a mutational path leading from one protein phenotype to another phenotype [9], [10], [15], [26], [27], [48], [49]. Phenotype is often defined in terms of biological function. Here we identify phenotype of a protein with its tertiary structure that underlies function. In the HP model we adopted, genotypes are modeled by polymer sequences consisting of 18 monomers that can either be hydrophobic or polar, whereas phenotypes are modeled by the conformations that these polymer chains can configure, as self-avoiding walks, on a two-dimensional (2D) square lattice [23], [29]. The simplicity of this setup allows for a complete, exhaustive description of the sequence-to-structure mapping within the model (see Methods). The availability of such a mapping is of immense benefit to the modeling of evolutionary processes [31]. Folded proteins in short-chain 2D HP models (with 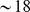 monomers) have ratios between inside and outside residues similar to those of three-dimensional real proteins of lengths 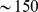 residues [29]. As a result, despite these models' limitations in capturing detailed energetics of protein folding [50], [51], short-chain 2D HP and HP-like models have been shown to embody general trends in the sequence-to-structure mapping of real proteins, exhibiting features such as a particular form of non-randomness in the distribution of hydrophobicity along real protein sequences [52], domain structure and autonomous folding units [30], [31], [53], and homology-like behaviors in the sequence-structure mapping [54].

In our terminology, *extended* neutral networks in our model refer to networks that include sequences that fold uniquely to a given structure *as well as* sequences that have the given structure as one of its multiple native structures, wherein native-state degeneracy 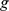 is limited to the range 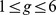 [23]. In other words, one native structure is shared by all of the sequences with 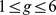 in an extended neutral network. In contrast, the *core* neutral network of a given structure refers to the more conventional network consisting only of 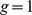 sequences that fold uniquely to that structure. Thus the core network is a subnet of the extended network for the same structure. In accordance with this definition, bridge proteins are found in extended networks, and a single bridge protein can belong to several extended neutral networks.

**Figure 1:**
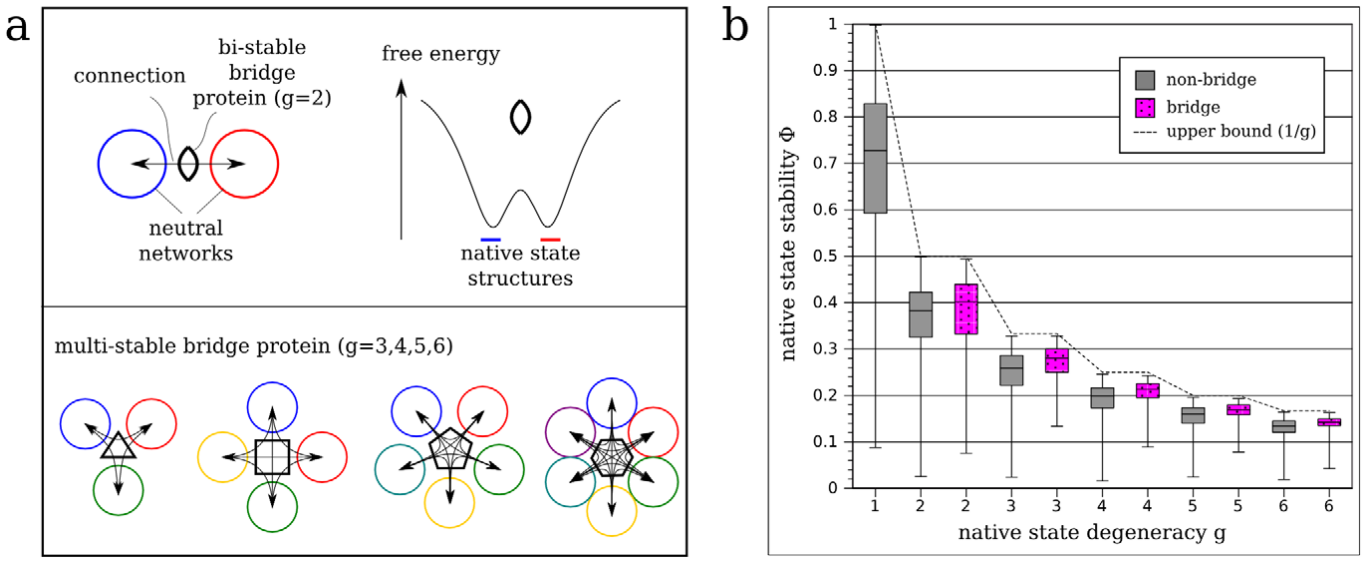
Bi-stable and multi-stable proteins acting as evolutionary bridges. (**a**) Proteins with degenerate native states (two or more structures with maximum stability) can function as connectors between neutral networks. Bi-stability is indicated for a bi-stable protein by a schematic folding funnel with two free energy minima. In an ideal case, the neutral network of sequences for each native-state structure can be reached by a single mutation from a centrally located sequence. This would enable efficient evolutionary transitions between those neutral networks. The frequency of such a bridge protein sequence may be maintained at an appreciable level in populations evolving under adaptive conflicts. Bridge proteins with up to six-fold native-state degeneracy (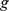) are illustrated. (**b**) Box plots of native-state stability (i.e., fractional population 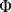) versus native state degeneracy (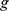) of all model sequences with 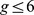 (grey), and the subset of all bridge sequences (magenta). Here we follow the standard convention for box plots in descriptive statistics: For each sample defined by a given value of the variable plotted along the horizontal axis (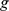 for bridge or non-bridge sequences in this case), the lowest and highest bars are, respectively, the lowest and highest values of the property of interest (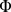 in this case) observed for the given sample. The top and bottom of the box are, respectively, the corresponding lower and upper quartiles of the sample, whereas the median of the sample is shown by the line within the box. The box plots here indicate that bridge sequences are significantly more stable than non-bridge sequences (Wilcoxon Rank Sum Test; 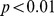 in all cases, except 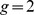, where 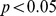). In the biophysical protein chain model used here (see Methods), the upper bound of 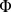, given 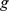, is 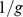 (black dashed line).

For a protein with native-state degeneracy 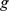, it can serve to connect a maximum number of 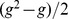 pairs of core neutral networks among the 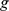 neutral networks defined by the protein's 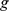 native structures (see Figure 1a and entries along the sixth row in Table 1). Specifically, a two-network connection is effected by a 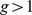 sequence if there is a single-point mutation that can result in a sequence in one core neutral network while there is another single-point mutation that can result in another sequence that belongs to another core neutral network. Likewise, connections can also exist between more than two networks, if the 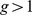 sequence can be mutated into 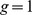 sequences of more than two core networks. Sequences that can effect such connections are referred to as bridges. Exact enumeration of all model sequences with 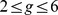 shows that 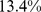 of these sequences are bridges with at least *one* connection (i.e. linking at least *two* neutral networks; see Table 1). All other 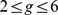 sequences — which are not bridges — can only be mutated to a 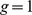 sequence from only one core neutral network, or alternatively cannot be mutated to a 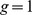 sequence at all.

**Table 1:** Bridge proteins as connectors in sequence space.

native-state degeneracy 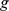 of multi-stable protein	2	3	4	5	6	all 2,…,6
**number of multi-stable proteins in HP model**	11018	6212	8541	5193	6690	37654
**number of bridges (** 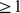 ** neutral network connection)**	1421	1088	967	852	721	5049
**percentage of bridges among multi-stable proteins**	12.897%	17.514%	11.321%	16.406%	10.777%	13.408%
**percentage of bridges in sequence space**	0.542%	0.415%	0.368%	0.325%	0.275%	1.926%
**upper limit of neutral network connections per bridge ** 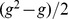	1	3	6	10	15	n/a
**observed neutral network connections per bridge (average)**	1	1.449	1.774	1.959	2.008	1.551
**percentage of bridges reaching upper limit of neutral network connections**	100%	22.426%	5.791%	0.469%	0.693%	34.264%

A bi- or multi-stable protein (with a degenerate native state) is a bridge sequence if it has at least two 1-error mutants that fold non-degenerately into at least two different structures among the sequence's multiple native-state structures, i.e., each mutant folds uniquely to a different structure. In other words, there is at least one connection to the core of each of the two or more neutral networks. In total, for sequences with chain length 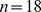 studied here (see Methods), there are 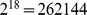 sequences in the model sequence space, 6349 of which have non-degenerate (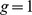) native states.

Bridges constitute almost 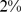 of the entire sequence space in the present model (Table 1). Interestingly, this percentage is comparable to the 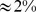 of all sequences that fold uniquely, i.e., have 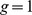 in the same model [55]. While the true scaling factors that relate the 2D lattice model to real proteins are unknown, one can speculate that the sequence space of real proteins could also exhibit similar proportions of uniquely folding proteins vs bridge proteins.

Results in Table 1 also show that only a small fraction of the possible maximum number of connections was achieved by most of the bridges in the HP model. For instance, a bridge with six degenerate native-state structures (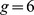) could connect up to 15 pairs of the six associated neutral networks. However, the average number of connections among such bridges is only about two (second last row in Table 1), even though there is a small percentage of bridges that can realize the maximum number of connections (bottom row in Table 1). In view of these statistics and for the sake of terminological simplicity, we will use the term bi-stable below regardless of whether or not the protein has 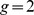 or 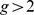 when the meaning of the term is clear from the context of the discussion.

#### Increased connectivity of extended neutral networks

Having identified bridge proteins among model proteins, we now study the potential advantage of bridges for protein evolution due to the bridges' ability to provide additional viable pathways through sequence space. When comparing all 17205 pairwise combinations of the 186 extended neutral networks (with five or more sequences per core network), 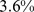 of these combinations shared at least one bridge. The percentage of one-mutation connections between neutral networks increased considerably (from 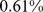 to 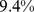) when extended networks were included, instead of only the core networks. Among the 22 largest networks (with 20 or more sequences per core network), the percentage of the 231 network pairs sharing a bridge was even higher (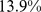). Likewise, the percentage of one-mutation connections between core and extended networks increases from 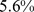 to 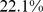. Large neutral networks are of particular importance for molecular evolution [56] because they provide a higher degree of mutational robustness and thus are more likely to be populated over long time scales when compared to smaller neutral networks with lower mutational robustness [57], [58]. A neutral network resembles a protein family. In this regard, a large network could allow more variants among a protein's descendant to be functional. Now our model results indicate that a large network probably enjoys an added advantage of enhanced evolvability by virtue of its increased connectivity to other networks.

#### Bridge proteins have relatively stable native states

Another factor that makes bridge proteins form viable connections between neutral networks is their significantly higher median native state stability (measured by the fractional population 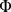; see Methods) compared to proteins with the same 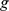 but that are not bridges (Figure 1b). This phenomenon can be readily explained by the close sequence space adjacency of bridge proteins to the prototype sequences of neutral networks (see Figure S1 in the supporting information online). A prototype sequence is the most thermodynamically stable protein within a neutral network. Since native stability increases with decreasing Hamming distance from the prototype [23], bridges are on average more stable than non-bridge sequences because they are closer to the prototype.

#### Potential bridge proteins in the Protein Data Bank

In contrast to the complete account of bridge proteins in a simple model that we obtained by exact enumeration, it is currently not feasible to achieve the same for actual proteins. Nonetheless, we may scan the available data on proteins to identify candidates that have a high likelihood of bi-stability by using the broadest criteria for a bridge protein, viz., the existence of two distinct structures with similar stabilities that are encoded by the same amino acid sequence. A potential source of such candidates that has recently become available is the Protein Conformational Database (PCDB) [59] that collects all instances of proteins in the Protein Data Bank (PDB) for which more than one structure has been experimentally determined. Because many of these instances could have been caused merely by experimental uncertainties, especially when there is only a small backbone RMSD (root mean square deviation of backbone atoms) between the alternate structures, we only considered proteins with an RMSD of at least 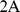, which has been considered to indicate a substantial structural difference [60]–[62], and structure pairs that shared 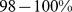 sequence identity. Once a protein was so identified, Rosetta free energy scores [63] (corrected for differences in protein chain length; see Methods) were computed for both structures of the protein, and a small difference between these scores was taken to be a measure of high bi-stability. In the present investigation, only those proteins that have Rosetta-determined stability differences among the smallest 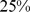 of all stability difference values in the initial set of candidates screened by the 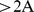 criterion were considered as potential bridges. We consider the resulting subset of candidates after two levels of screening to have satisfied rather stringent structural and energetic requirements for bridge proteins, and thus may serve as starting points for further experimental investigations of possible bi-stable behaviors. A list of these proteins is provided in Dataset S1. Our dataset contains only single-domain proteins because PCDB only compares structures of single-domain (i.e., not multi-domain) proteins as defined by CATH [64].

A selection of these putative bridge proteins is highlighted in Table 2. This selection comprises the ten candidates with the smallest stability difference and the ten candidates with the highest RMSD between structure pairs (Nitrophorin-4 belongs to both categories), as well as two proteins that have previously been shown experimentally to exhibit bi-stability (the transcriptional regulator Rop [65] and Cytochrome P450 [66]). Lymphotactin, which is included in Table 2, has previously been found to be bi-stable, forming two very distinct structures [67]. However, the automated clustering in PCDB did not pair these distinct structures. Instead, it paired different versions of the same structure, which has a long disordered tail. This is an illustration of the potential limitations of simple automated approaches. At the same time, this example also reflects the challenges that bi-stable proteins pose for experimental methods that often require significant modifications of a protein and its interactions (e.g., changes in the protein's amino acid sequence, binding partners, or environmental conditions) in order to capture their alternative structures.

**Table 2:** Examples of proteins with putative high bi-stability.

accession	protein names	organism	domain pair^a^	domain length	RMSD^b^	sequence identity (%)	average stability^c^	stability difference
Q8WTS6	Histone-lysine N-methyltransferase SETD7	*Homo sapiens*	1h3iA01 1mt6A01	134	3.53	98.53	−3.16797	0.00021
Q9BMI9	Purine-nucleoside phosphorylase	*Schistosoma mansoni*	1tcvA00 1td1B00	271	2.16	98.18	−3.26465	0.00046
P01854	Ig epsilon chain C region	*Homo sapiens*	1fp5A02 1o0vB03	108	2.06	98.18	−2.93154	0.00063
P54939	Talin-1	*Gallus gallus*	1mixA02 1mk7B02	93	2.12	100.00	−2.96602	0.00131
P00183	Camphor 5-monooxygenase (Cytochrome P450-cam)	*Pseudomonas putida*	1k2oA00 1gjmA00	429	2.13	99.75	−3.13376	0.00176
Q94734	Nitrophorin-4 (NP4)	*Rhodnius prolixus*	3c78X00 2ofmX00	184	13.09	100.00	−3.22443	0.00204
P00489	Glycogen phosphorylase (Myophosphorylase)	*Oryctolagus cuniculus*	2pyiA01 2gpaA01	476	4.08	99.38	−3.39796	0.00251
Q63537	Synapsin-2	*Rattus norvegicus*	1i7nA03 1i7lA03	119	2.15	100.00	−3.08808	0.00266
Q08012	Protein enhancer of sevenless 2B	*Drosophila melanogaster*	2a36A00 2azsA00	59	2.34	100.00	−2.51793	0.00345
P0A0N4	HTH-type transcriptional regulator qacR	*Staphylococcus aureus*	2g0eA02 1jumA02	137	2.67	100.00	−3.36183	0.00366
P56210	50S ribosomal protein L11 (Fragment)	*Bacillus stearothermophilus*	1foxA00 2fowA00	76	6.71	100.00	−2.67946	0.00875
P98170	Baculoviral IAP repeat-containing protein 4	*Homo sapiens*	1f9xA00 1tfqA00	117	10.59	100.00	−2.36664	0.00994
P03051	Regulatory protein rop	*Escherichia coli*	1b6qA00 1gmgA00	56	2.24	100.00	−3.34805	0.01144
P69441	Adenylate kinase	*Escherichia coli*	4akeA00 2eckA00	214	7.2	100.00	−3.39316	0.01445
Q7SIG1	Hydrolase	*Alicyclobacillus acidocaldarius*	1u4nA00 1qz3A00	308	8.64	99.68	−3.36802	0.01768
P01008	Antithrombin-III (Serpin C1)	*Homo sapiens*	1jvqL01 2gd4C02	140	17.16	100.00	−2.58988	0.02646
P47992	Lymphotactin (C motif chemokine 1)	*Homo sapiens*	1j8iA00 1j9oA00	93	11.77	100.00	−2.09003	0.02818
P69541	Capsid protein G8P (Gene 8 protein)	Enterobacteria phage M13	2cpbA00 2cpsA00	50	8.53	100.00	−2.90853	0.03448
Q55080	Cytochrome P450 119 (Peroxidase)	*Sulfolobus acidocaldarius*	1io9A00 1f4uA00	366	2.16	100.00	−3.36023	0.03841
P0C0Y1	Light-harvesting protein B-875 beta chain	*Rhodobacter sphaeroides*	1dx7A00 1jo5A00	48	10.11	100.00	−2.69183	0.04678
P83917	Chromobox protein homolog 1 (Heterochromatin protein 1 homolog beta)	*Mus musculus*	1ap0A00 1guwA00	73	7.42	100.00	−2.30409	0.05826

Proteins are ordered by increasing stability difference (decreasing bi-stability) and they may be classified into the following functional categories: metabolism (Purine-nucleoside phosphorylase, Camphor 5-monooxygenase, Glycogen phosphorylase, Adenylate kinase, Hydrolase, Cytochrome P450 119, Light-harvesting protein B-875 beta chain), transcriptional regulation (HTH-type transcriptional regulator qacR, Regulatory protein rop), epigenetic regulation (Histone-lysine N-methyltransferase, Chromobox protein homolog 1), signalling (Synapsin-2, Protein enhancer of sevenless 2B, Baculoviral IAP repeat-containing protein 4, Antithrombin-III, Lymphotactin), translation (50S ribosomal protein L11), cytoskeleton (Talin-1, Synapsin-2), and host-pathogen interaction/immune system (Ig epsilon chain C region, Nitrophorin-4, Baculoviral IAP repeat-containing protein 4, Lymphotactin, Capsid protein G8P).

a CATH [64] structural domain identifiers.

b Root Mean Square Deviation of backbone atoms; in units of Å.

c Rosetta standard free energy score/domain length.

The proteins in Table 2 perform a variety of biological functions. Some of the listed examples are metabolic enzymes, others are involved in epigenetics, transcriptional regulation and signaling, larger intra-cellular structures, or immune functions. It will be interesting to investigate possible roles of bi-stable structural dynamics in these proteins' function. Often bi-stability is a part of a protein's normal functional dynamics. A common example is the conformational changes that some proteins undergo upon binding to a ligand. In that case, the alternate structures are associated with the same “native” function. Such structural dynamics is unlikely to be an evolutionary response to adaptive conflicts. Nevertheless, such bi-stable behavior may, under certain evolutionary conditions, lead to one of the protein's native functional/structural states being co-opted for a different function. In that case a situation of adaptive conflict would be created.

### Evolutionary landscapes of bi-stability around bridge proteins

#### Smooth bi-stability gradients around bridge proteins

As discussed above, our model suggests that only a small fraction of the sequence space are bridge proteins (Table 1). This phenomenon raises the question as to the relevance of bridge proteins to evolution, because it would appear that the likelihood of these proteins emerging by random drift is small. However, evolution toward new protein phenotypes does not have to rely solely on random drift. It can be a directed, adaptive process. In this regard, model simulations have shown that evolving proteins can respond to selection of an excited (i.e. non-native) state structure before mutations convert the protein sequence from one that folds to a given original native structure to another sequence that folds into a new native structure [32]. As a consequence of the sequence space energy funnels around prototypes (see above), the stability gradient, i.e., the variation of stability with respect to change in sequence, for any given protein structure is essentially smooth [23], [68]. In this perspective, because bridge proteins lie in between two neutral networks — i.e. they reside in an overlap region of two sequence space stability funnels — mutations in a bridge protein are expected to gradually stabilize one structure and destabilize the other.

**Figure 2:**
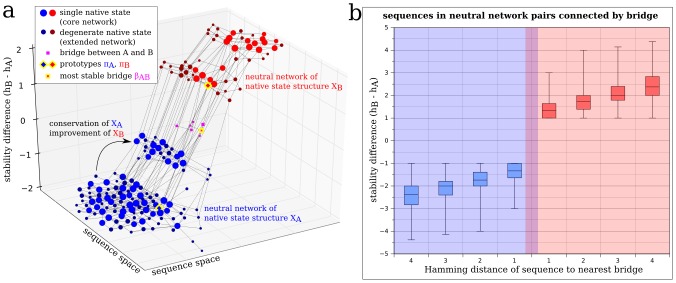
Bi-stability decreases with increasing sequence distance from bridge proteins. (**a**) An example of the distribution of bi-stability in a small section of a model sequence space. The difference in the number of hydrophobic contacts, 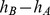, (stability difference) for the native-state structures 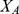 and 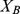 of two adjacent neutral networks 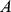 and 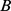 (blue and red, respectively) are depicted by a two-dimensional representation of sequence space (see Methods). Nodes represent sequence variants. Node sizes are scaled according to native-state stability (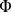, a larger node size corresponds to a large 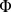 value). Edges connect sequences that differ by one mutation. The arrow indicates a mutation from a sequence with a stability difference of 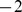 to a sequence with a stability difference of 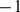. In other words, this mutation increases stability for 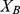 while conserving 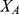 as the native state. Bridge proteins (magenta squares) are equally stable for both native states and thus have a stability difference of zero. (**b**) Generalization of smooth bi-stability gradients around bridge proteins. Each box plot gives the distribution (i.e. the entire data range with vertical lines delimiting quartile boundaries as specified in the caption for Figure 1b above) of 623 average stability differences computed for individual sequences that belong to the same neutral network and can be mutated into a bridge protein with the same given number of mutations (i.e. have the same Hamming distance from a bridge). The stability difference was calculated between the native structures of all 623 pairs of extended neutral networks (that have at least 5 core nodes, and at least one bridge). Data for each pair was counted only once, and the color blue is used in this plot for the larger network of each pair. The further away a sequence is located from a bridge in sequence space, the higher its stability difference towards one of the two structures, and the lower its bi-stability. All differences between box plots were significant (Wilcoxon Rank Sum Test, 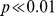).

To elucidate this expected trend, we used our model to measure the stability difference 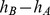 between two structures, 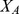 and 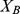, for all sequences in the corresponding neutral networks A and B (Figure 2 and S2), where 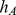 and 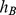 are the number of intra-chain hydrophobic-hydrophobic (HH) contacts (the only type of interactions that carries a favorable energy in the model; see Eq. 1 and 2 in Methods). This metric reflects the propensity of a protein to fold into one or the other structure, and thus reflects the degree of bi-stability: the more similar the stability values, the higher the degree of bi-stability, and vice versa. It follows that the highest degree of bi-stability in our model is reached when the native state is degenerate (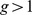), in which case the stabilities of the multiple native structures are exactly equal. A negative stability difference, 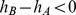, corresponds to 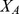 as the most favorable sole native structure, whereas a positive value corresponds to 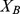 as the most favorable sole native structure. Both structures are equally stable for a bridge protein. Hence bridge proteins have a stability difference of zero (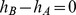). Within a given neutral network, the stability difference between the native structure of the neutral network and the native structure of an adjacent neutral network can vary (Figure 2a). Therefore, an evolving protein can increase its stability toward a nonnative structure, while still maintaining its original structure as the sole native (most favorable) conformation.

We have quantified the gradual bi-stability change around bridge proteins in our model by considering all non-redundant pairs of extended neutral networks, each with at least five core sequences, wherein the two networks in each pair are connected by at least one bridge sequence (Figure 2b). A clear correlation is seen between the degree of bi-stability and the Hamming distance (number of mutational steps) of a sequence from the nearest bridge.

#### A bi-stability gradient along an experimentally determined mutational path

Inspired by the finding of the gradual sequence-space distribution of bi-stability in our simple biophysical protein chain model, we applied the methodology developed above to study the bi-stability of experimental protein structures. As a first step, we conducted an analysis of a set of sequences discovered by Alexander et al. [27], [69], who have mutationally inter-converted the albumin-binding (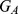) and immunoglobulin-binding (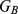) domains of *Streptococcal* protein G — two structurally very dissimilar domains — by introducing a series of single point mutations that do not change the respective native structures until one critical mutational step in the interconversion. One pair of single mutations (forward and reverse) inter-converts between two sequences (labeled 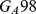 and 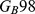) that predominantly encode either for one native structure or the other; and a small degree of measurable bi-stability was observed in the 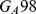 mutant [27]. Recognizing that this experimental observation comes remarkably close to the situation envisioned by our theoretical investigations, we attempted to verify a gradient of increasing bi-stability toward the bridge state (i.e. the structural switch) similar to that predicted above by our model.

**Figure 3:**
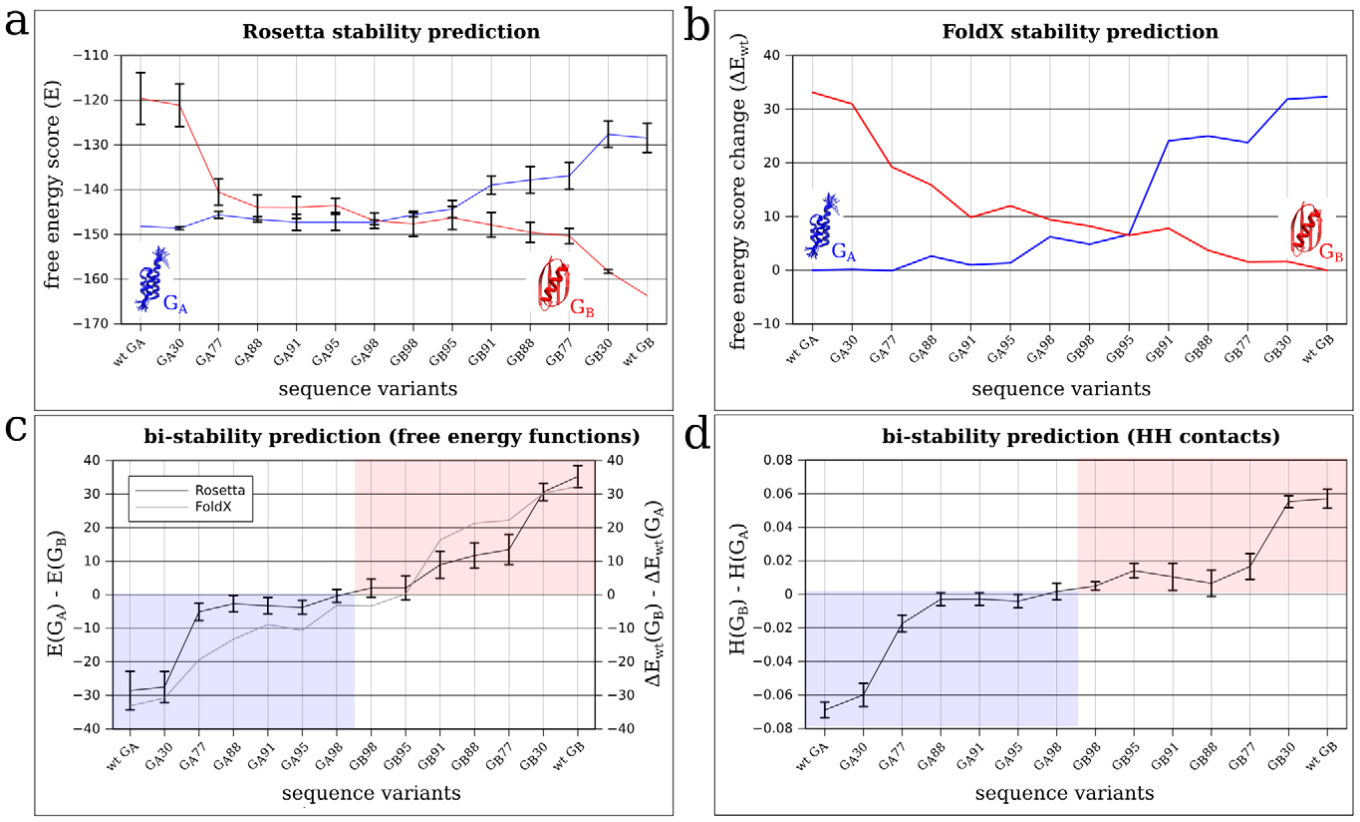
Modeling the folding stability of an experimentally determined mutational path from one real protein structure to another indicates a gradual stability shift. In this study, the PDB structures of 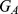 (PDB code 2FS1) and 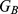 (PDB code 1PGA) were used as wildtypes (wt) for energy calculations and mutagenesis. There is only 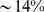 sequence identity between wt 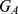 and wt 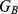 sequences. Based on the 2FS1 and 1PGA structures, the structures of all intermediate mutants (sequence variants) were constructed by either FoldX or Rosetta in accordance with the published sequences in Alexander et al. [27], [69]. The sequence labels along the present horizontal axes have the same meaning as those in these references. (**a**) The average free energy (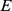) predicted by the standard free energy scoring function of Rosetta was computed for all sequence variants. The 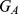 and 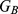 structures of the sequence variants were also constructed by Rosetta with its FastRelax free energy minimization procedure. The plotted averages (connected by lines that are merely a guide for the eye) were obtained from 25 replicate calculations and all error bars in this and other panels of this figure correspond to one standard deviation from the mean. (**b**) The FoldX free energy scores relative to that of the wildtype (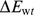) for all 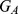 and 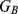 variants. (**c**) Bi-stability of each sequence variant is quantified by the difference in predicted free energy between the variant 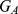 and 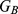 structures (vertical axis). Free energy differences are computed using either the Rosetta values in (a) (black line with error bars) or the FoldX values in (b) (grey line). (**d**) Here bi-stability of each sequence is quantified by the difference in hydrophobic contact density 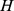 (see Methods) between the Rosetta/FastRelax-constructed structures of 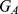 and 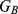 for the given sequence (same structures as those in (a)). In (**c**) and (**d**), the blue area covers sequences that are known experimentally to adopt 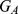 as their native structure, whereas the red area covers sequences that are known experimentally to adopt 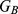 as their native structure.

To this end, we used Rosetta [63] and FoldX [70] (see Methods) to insert the mutations determined by Alexander et al. into the wildtype structures of 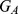 and 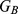, with the intermediate labels in Figure 3a–d (horizontal axes) reflecting sequence identity, in accordance with the notation in Ref. [27]. For instance, the sequence pairs 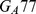 and 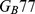 share 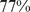 sequence identity. For each sequence variant, the relative favorability of the two structures was computed using Rosetta or FoldX. Structure 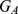 is the favored native state on the left side of Figure 3a–d: it has a higher stability (a lower energy) than 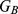. In this situation, 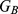 is interpreted as an excited state. The left-most variant labeled “wt 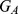
” corresponds to the wildtype sequence for 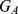. The roles of 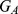 and 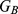 are reversed on the right side of these plots, with structure 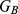 now being the favored native state.

Figure 3a shows the absolute free energy scores (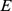) computed with Rosetta. The resulting trend demonstrates that this tool is able to accurately predict which of the two structures is the native state (more stable, more negative free energy scores) for each of the sequences.

The FastRelax [71] method of Rosetta in combination with strong Lennard-Jones repulsions [72] was used for free energy minimization for all Rosetta applications in the present study. Because FastRelax allows for movements of the side chains and the backbone, and can therefore produce multiple similar but nonidentical structures for the same sequence variant, the averages and standard deviations of the free energy scores determined from 25 replicate calculations are given in Figure 3a to provide a more comprehensive presentation of the Rosetta prediction.

In contrast to the Rosetta/FastRelax method, FoldX fixes the main chain and only optimizes the side chains. A possible consequence of this limitation is that the absolute free energy scores (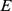) of the wildtypes were rather unfavorable and differed considerably between 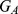 (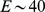) and 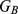 (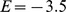). Assuming that FoldX in conjunction with side chain optimization reasonably models the relative stabilities of different sequences adopting the same given main-chain structure even though the FoldX protocol may not account for the relative stabilities of different main-chain structures, Figure 3b shows only the relative change of free energies, 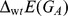 and 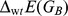, from the respective wildtype. The trend of gradual change in stability with respect to sequence variation along each of the two individual curves (for 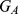 and 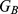 respectively) is similar to that obtained using Rosetta, but the FoldX free energies themselves cannot capture the expected structure switch between 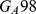 and 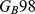.

Based on the results in Figure 3a,b, Figure 3c shows the stability differences, 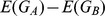 (Rosetta) and 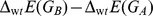 (FoldX), with the convention that negative differences correspond to 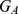 being the favored native state (light blue area in Figures 3c,d). This representation of the data allows for a more direct visual comparison with Figure 2, where model proteins were shown to follow a similar trend of increased stability for an excited, non-native structure, while maintaining the same original native structure, as the point at which the mutated protein undergoes a structure switch is approached.

A very similar trend was observed in Figure 3d, where instead of employing the complex free energy functions of Rosetta and FoldX, we applied a simpler measure of stability, 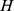, defined for any given sequence with respect to a given structure as the fraction of inter-residue contacts that are between carbon atoms among all atomic contacts (see Methods). Thus this measure may be viewed as a hydrophobic contact density. It corresponds roughly to the number 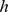 of HH contacts in the simple HP model normalized by the total number of contacts (i.e., 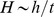, where 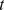 is the total number of nearest-neighbor topological contacts in the lattice chain model [73]). The normalization was introduced to facilitate comparison of the 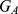 and 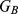 structures on a more equal footing in view of their considerably different total numbers of atomic contacts due to the presence of disordered termini in 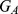 but ordered termini in 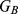. Accordingly, 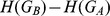 is defined as a measure of stability difference between the two structures.

As in the computational results in Figure 3, the overall native stability of the mutants measured experimentally by Alexander et al. decreases with decreasing sequence distance to the structure switch between 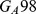 and 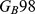 [27]. Indeed, this observation is expected in the conceptual framework of stability super-funnels [23] and with the low native-state stability of bridge proteins (Figure 1b). A recent study on HP model proteins of sequence length 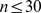 [74] based on an efficient algorithm [75] has also made the observation of decreased stability around the borders of adjacent neutral networks, although this study focused only on 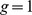 sequences and did not address bi-stability.

### Evolutionary population dynamics under adaptive conflict

#### Bi-stable proteins dominate the steady-state population under weak adaptive conflict

To better understand the potentially important role of bi-stability in evolution under conflicting selection pressures (adaptive conflicts), we have also performed evolutionary simulations of sequence populations under two selection pressures. Each sequence of the combined neutral networks in Figure 2a was assigned a fitness value based on their stability for the two beneficial native-state structures 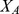 and 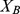 (see Methods). As a first step in this endeavor, we carried out deterministic population dynamics simulations where initially only the prototype of network A (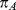) was populated. This corresponds to a scenario in which previous strong purifying selection has eliminated all but the most stable protein variant of all the proteins that encode uniquely for structure 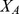. The simulations began with the onset of a second selection pressure, for 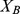, that is as strong as the selection pressure for 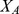. This created an adaptive conflict to simultaneously conserve 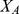 and also to improve stability for 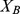. Subsequent iterations of the master equation (Eq. 5) allowed the spreading of fractional sequence population 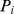 from 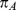 towards all other allowed sequences 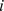 (i.e., all sequences plotted in Figure 2a), representing the evolutionary change within an infinite-size population over time. The spreading of the population over all allowed sequences was determined by two main factors: 1) the fitness distribution among sequences; and 2) the neutral network connectivity.

**Figure 4:**
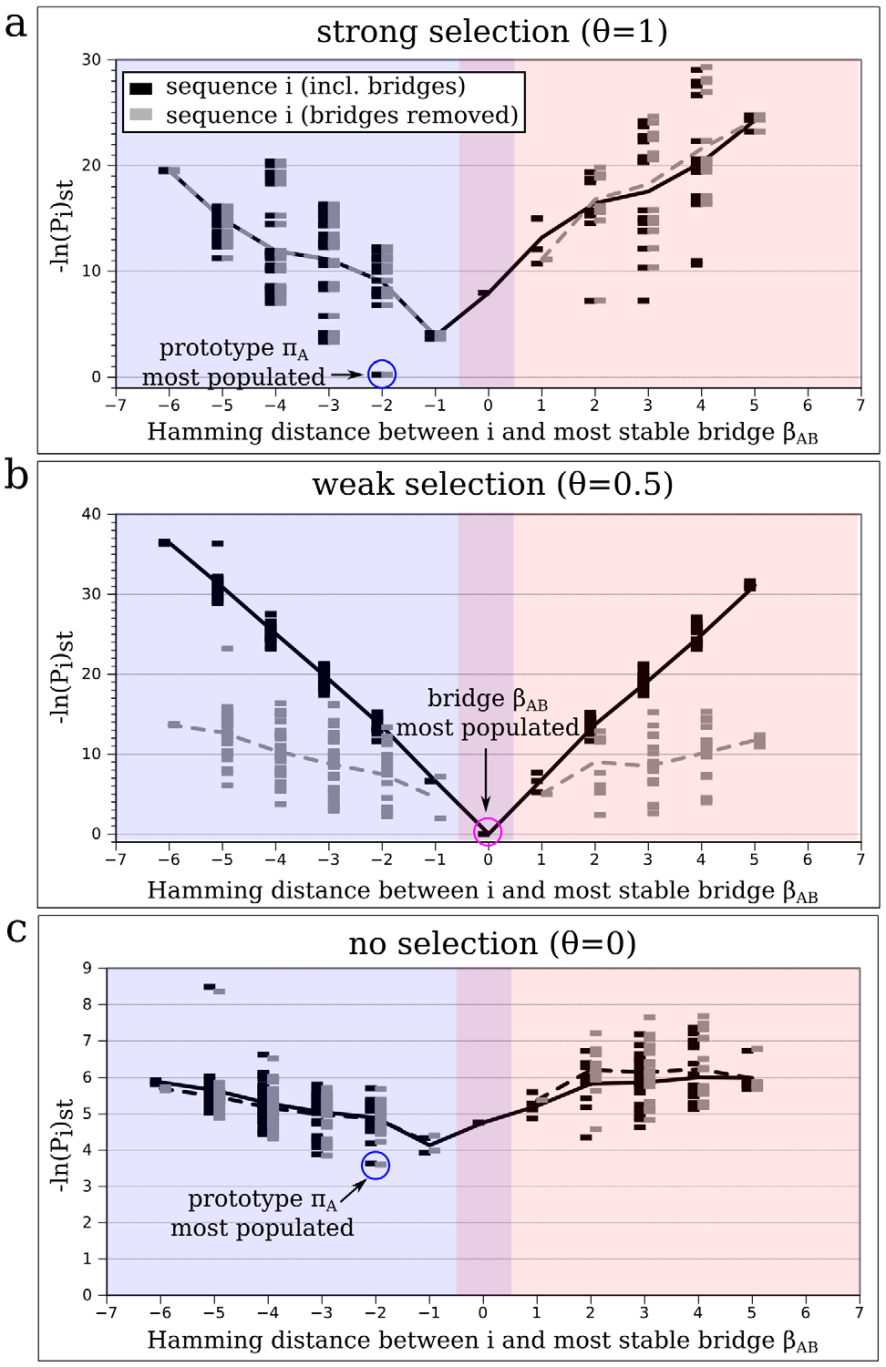
Steady-state populations of protein sequences after simulated evolution under adaptive conflict. The negative logarithmic steady-state populations, 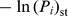, of all sequences 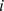 from two adjacent extended neutral networks (A in blue, B in red, overlap region in magenta; see Figure 2a) in our biophysical protein chain model are plotted against their respective Hamming distances with respect to the most stable bridge protein sequence 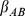. A low 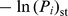 value corresponds to a high population (probability) at steady state. The 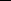 and 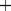 signs of the sequence distance values (horizontal axis) distinguish between the two networks A and B, respectively. Steady state populations were obtained with (black symbols) and without (grey symbols) bridge proteins for an example neutral network pair, under three different selection pressures: 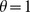 (**a**; strong selection), 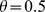 (**b**; weak selection), and 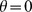 (**c**; no selection). The same selection pressure was applied for both selected structures (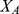 and 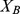). The black solid and grey dashed lines indicate the average 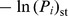 values as functions of Hamming distance that were simulated, respectively, with and without the bridge sequences. To facilitate comparison, even in cases where bridge proteins were removed from the neutral networks during the evolutionary simulation, Hamming distances are still defined by that between a given sequence and (the removed) 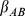.

The selection pressure 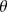 in our model parametrizes how much stability loss is tolerated upon mutation in either of the two structures, ranging from a maximum stability requirement (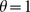; *strong selection*), over a relaxed stability requirement (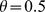; *weak selection*), to no specific stability requirement (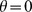; *no selection*, as long as 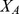 and 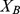 were among the native states — otherwise the sequence was considered non-viable). The steady-state population 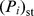 (plotted as the negative natural logarithm 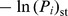 in Figure 4) represents the frequency, or probability, of a certain sequence 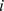 at steady state, i.e. when the evolutionary dynamics had resulted in a time-independent population distribution. The definition of 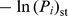 is akin to that of free energy in statistical physics, with more negative values for 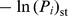 representing higher populations. This choice of sign convention is in accord with the super-funnel [23] and “mortality landscape” [30] imageries that invoke a “downward” driving force toward the more favorable (i.e., more populated) regions (attractive basins) of the sequence space [51]. Here, the quantity 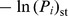 is plotted against the Hamming distance of 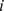 from the most stable bridge sequence, 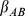. As a control, steady-state populations were also calculated for an altered sequence set, where all bridge proteins were removed. The purpose of this control was to establish the relative importance of bridge proteins compared to other bi-stable proteins (such as the ones summarized in Figure 2b) under adaptive conflict.

Under strong selection (Figure 4a), the prototypes of each neutral network — not the bridge proteins — have highest fitness. Because the network on the left (blue) has the more stable prototype, steady-state population accumulated there (lowest 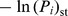 at Hamming distance −2 from 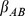). In the absence of fitness selection (Figure 4c), steady-state populations are only determined by network adjacency/topology [21], [23] in that sequences with many neutral neighbors in the network are more populated at steady state, regardless of how stably they fold into their native structure. A very similar steady-state population distribution to the previous strong-selection case was observed, owing to a high correlation between mutational robustness (i.e. neighbours in the neutral network) and thermodynamic stability [23], [45], [68], [76], [77]. Prototypes generally exhibit both properties prominently [22]. In both the “strong selection” and “no selection” cases, steady-state populations are largely unaffected by the removal of bridge proteins from the networks (open symbols).

In contrast, a very different steady-state distribution emerged under weak selection (Figure 4b). In this scenario, the most stable bridge protein (magenta circle) is clearly favored over any other sequence (black symbols). This distribution was also preserved, albeit to a lesser degree, when the actual bridge proteins were artificially removed (grey symbols) so as to force the evolutionary process to populate other sequences in this control simulation. The population as a whole is more equally spread out over many sequence variants, but it is still concentrated around the intersection zone between the two neutral networks (where bridges are usually located). Bridge proteins, despite being highly advantageous, are therefore not absolutely necessary for the evolution of bi-stability. The *bi-stability landscape* in Figure 2 helps to explain why model proteins that are close to bridge proteins in sequence space are more populated when bridges are removed: they are the next best solution to providing the two beneficial structures. Instead of equal stability for 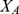 and 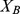 (in bridge proteins), one of the two structures is more stable than the other (by as little as one hydrophobic contact) in these model proteins. This stability difference is still large enough, however, to incur a considerable reduction in fitness — and thus steady-state population — compared to a bridge protein.

To assess the generality of the trends revealed in Figure 4, we repeated the master-equation evolutionary dynamics simulation for several additional neutral network pairs. The resulting steady-state populations (Figure S3) exhibited trends very similar to that in Figure 4, i.e., a V-shaped distribution of 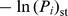 around 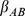 under weak selection pressures and a considerably flattened distribution upon the removal of bridges.

We have also examined how evolutionary steady states are achieved in our model. Under weak selection, the model evolutionary dynamics prior to achieving steady state indicates that the population of the initial prototype sequence 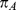 decreases gradually as it spreads to mutants within network A, resulting in other 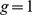 sequences being populated (Figure S4a; solid blue lines). At the same time, the highly beneficial bridge sequence 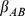 increases rather quickly to become the highest-populated genotype, giving rise to the steady-state population distribution in Figure 4b. These results were first obtained using our master-equation (ME) method, which is an efficient approach to obtain the steady-state distribution. The ME describes the deterministic dynamics of an effectively infinite population that can access all possible sequences. Recognizing that this assumption may be biologically unrealistic except for “quasi-species”-like systems consisting of fast-replicating entities such as viruses [78]–[80], we sought to contrast the behaviors observed in our ME treatment with those obtained from stochastic Monte Carlo (MC) simulations of finite populations of 1000 individuals (Figure S4a; dashed lines; MC protocol described in Methods). Naturally, because of their stochastic nature, individual MC simulation runs differ. Nonetheless, when results are averaged over 100 simulations, a clear agreement in the general behaviors between the ME and MC simulations is seen, with both sets of simulations achieving essentially the same high steady-state population for the 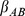 bridge (Figure S4a). Not unexpectedly, the evolutionary process in the finite-population MC simulation is somewhat slower than that in the ME simulation (cf. solid and dashed curves in Figure S4a) because there are continuous population transfers between neighboring sequences in every generation in ME but the corresponding discrete population transfers do not necessarily occur in every generation in MC. The evolutionary dynamics of the control case in which bridge proteins are not allowed to be populated is considered in Figure S4b. Consistent with Figure 4b, non-bridge sequences from networks A and B rose to significant frequencies, with network A being generally more populated due to its larger size. Because prototype 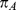 is more distant from the bridge sequences than prototype 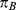 in terms of Hamming distance (2 vs 1) as well as stability difference (−2 vs 1; Figure 2a), 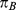 becomes more populated than 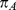 when 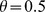 and bridges are not available (Figure S4b). Compared to the dynamics in Figure S4a with bridges, the time needed to achieve steady state takes considerably longer in Figure S4b when bridges are not available because no individual sequence possesses a strong fitness advantage to provide a strong drive for the evolutionary dynamics in this hypothetical case.

#### Bridge proteins persist under unequal selection pressures

**Figure 5:**
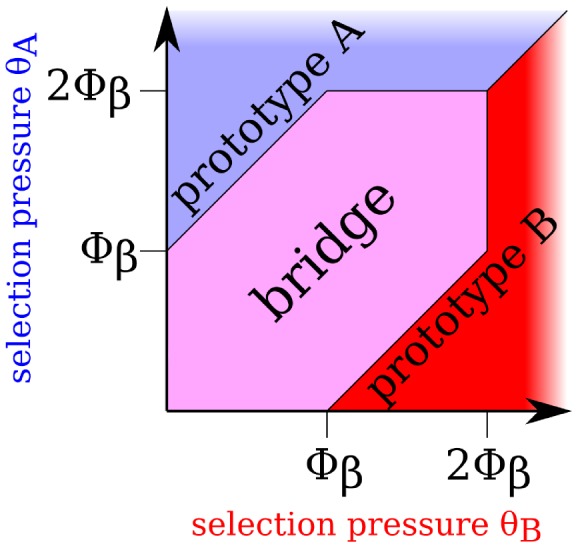
Bridge proteins persist under unequal selection pressures for two native-state structures. In our biophysical protein chain model, 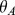 and 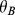 serve as the selection pressures on 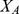 and 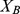, respectively, by setting the minimum required stability for optimal fitness. Here 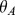 and 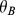 values are plotted in units of 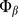, where 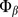 is the stability of the (equally stable) native-state structures (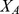 and 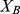) of the most stable bridge protein 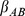. The magenta area is the range of 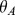 and 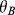 values within which bridge proteins have higher fitness than the specialized prototypes of neutral networks A and B.

To relate our model results to real proteins, we realize that it is unlikely that two selection pressures acting on the same protein are exactly equal. Indeed, the selection pressures may even change over time. To address the impact of such effects on our conclusions, we considered a model in which the selection pressures for two structures are not identical. Now, instead of using a single selection pressure 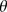 that applies to both structures 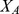 and 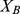 as in the above, we consider two independent selection pressures 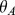 and 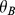. We found that the prominence of bridges is robust against imbalances between selection pressures under certain conditions (Figure 5). Intuitively, as is assumed in our model, the advantage of a bridge protein under adaptive conflict lies in the presumed additive nature of the fitness contributions derived from each native structure. Prototypes, on the other hand, are optimized for only one structure. Thus, essentially, they can only benefit from the fitness contribution from that one structure because fitness contributions from any other excited-state structure would be minuscule. If, in addition, there is a low requirement for native stability (i.e., low selection pressure 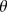), the advantage that a highly stable prototype (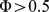; see Figure 1b) may have over the less stable (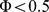) yet bi-structural bridge protein will be reduced even further. Only if the difference in selection pressures becomes too large, evolution will, as expected, then favor a more stable protein (e.g. prototype) for the structure under stronger selection. These trends are quantified in Figure 5. The magenta area at the center of the plot delimits the combinations of selection pressures that favor a bridge over a prototype, whereas the blue and red peripheral areas are dominated by a prototype.

## Discussion

Our analysis of an entire model protein sequence space demonstrates that viability of proteins with degenerate native states can confer an advantage under adaptive conflicts. In such situations, extensive overlaps exist between stability funnels of neutral networks, with bi-stable bridge proteins situated at the interface between networks. Although detailed characteristics of real protein sequence space remain to be elucidated, based on our model results we have little doubt that the investigation of bi-stability and evolvability is a promising area of future research. Bi-stability, however, cannot be the only evolutionary response to adaptive conflicts, because the two alternative conformations are mutually exclusive and thus the function of the protein can never be fully optimized. In this regard, the role of gene duplications would also be crucial. We leave this topic for another study [81].

### Strengths and limitations of the present simple biophysics model

Here we have employed a simple biophysical protein chain model to infer general properties of bi-stable proteins and their distribution in sequence space. The model used here is a simple exact model with an explicit representation of the protein conformations on a two-dimensional lattice. Despite their simplicity, such models capture essential features of the sequence-to-structure mapping of real proteins (see discussion in Results), and have provided significant insights into protein folding and evolution (reviewed in Refs. [31], [35]). The simple exact modeling approach allows a complete description of a system, but clearly such models are only a caricature of reality. In particular, only a limited variation of stability and bi-stability is allowed in our simple model, resulting in an appreciable percentage of sequences adopting two native structures with identical stability. Real proteins, in contrast, are unlikely to have exactly equal native stability in bridge proteins. Nonetheless, inasmuch as the goal of theoretical/conceptual models is to make predictions that can be tested experimentally, the main testable prediction of this work is that bi-stability can be increased or decreased by mutations leading either towards or away from bridge proteins, which are sequences that enjoy maximum bi-stability.

While the fraction of actual bridge proteins is unknown, one may speculate how the HP model relates to real proteins. For example, consider the following argument: Our simple model only allows for 10 different energy states (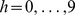 HH contacts). If the conformational ensemble of a real protein was mapped onto 10 equally sized bins of energy, the lowest-free energy bin (highest stability) could contain two structures with similar yet non-identical stabilities such that the protein may function as a bi-stable bridge (e.g. see Table 2). This relaxed definition of a bridge could entail that one structure would still be much more stable than the other (as in the case of 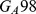 in Alexander et al. [27]). As a consequence, perhaps many such bridge proteins do not have easily measurable bi-stability because one structure remains dominant over the other. Nevertheless, the known examples of functional promiscuity suggest that even such unequal bi-stability may be of biological relevance. However, it is important to note that bi-stability can only occur if the two alternative native (or near-native) states are both thermodynamically accessible on time scales that are relevant for molecular functions.

The consequence of bi-stability landscapes (Figure 2) for evolution is that proteins evolving under adaptive conflict for two alternative structures (whose extended neutral networks are connected in sequence space) are automatically directed towards bi-stable states, and that the dynamics of this process do not have to rely entirely on random genetic drift. Bridge proteins may thus be created in the laboratory by providing appropriate combinations of selection pressures, or known bridge proteins can be stabilized towards one of their structural sub-states. So far, this gradual shift in bi-stability was studied in terms of structural phenotypes; but the same concept should also apply to other definitions of phenotypes that depend upon structural stability.

The simple fitness function in the present study rewards increased protein stability. This fitness function has provided significant insights; but it does not fully capture the subtle relationship between conformational stability and biological function in real proteins [82]. Too much stability can be detrimental for protein function, for example. More sophisticated biophysical models will need to be developed to incorporate such effects.

Future work should also improve the computational methods for determining bi-stability changes of *in-silico* mutated PDB structures. In this regard, the discrepancy between FoldX and Rosetta predictions in Figure 3a is noteworthy. Using these algorithms, only local structural optimization around PDB structures for 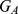 and 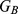 was performed in the present study. We made no attempt in global structural optimization, which amounts to using an amino acid sequence as the only input to determine its native structure, i.e., solving the protein folding problem for the given sequence. For this much more challenging task, scoring functions such as Rosetta that rely on comparative modeling have difficulties when presented with sequences that have a high degree of identity but fold to different structures nonetheless. The ability of Rosetta to arrive at the correct structure can be greatly enhanced by considering not only the amino acid sequence but also including experimental NMR chemical shift data as input [83], as has been demonstrated for the 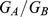 system [84]. This finding underlines that the scoring function alone is insufficient for this system. As emphasized recently by van Gunsteren and coworkers, the energetics that govern the structural transition between 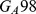 and 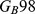 is highly delicate and cannot yet be accounted for atomistically using current force fields [85]. The quest for an accurate energy function for protein folding will likely remain a great challenge for years to come. In this light, the Rosetta criterion we adopted to obtain the present protein conformational diversity dataset (Dataset S1) is, inevitably, tentative. Nevertheless, based on the theoretical framework we developed and the general trend observed here, this dataset should serve as an impetus and provide useful candidates to be evaluated by future experimental investigations.

Our evolutionary simulations (Figures 4, S3, and S4) are idealized scenarios that do not realistically capture evolution in natural populations, where usually only a small portion of sequence space would be explored by individuals within a population that are related to each other by common ancestry. Our master equation approach and the calculated steady state therefore only give a general evolutionary trend: given enough time and mutations, a population will acquire the most bi-stable proteins. Nevertheless, we have shown that the nature of bi-stability landscapes (Figure 2) – where incremental shifts of excited state stability can lead towards increased bi-stability – have the potential to speed up adaptation under adaptive conflict, whenever such stability shifts are advantageous. Evolutionary experiments will be needed to test these predictions under natural conditions.

### Consequences for the theory of neutral networks

The increasing knowledge of promiscuous enzymes and the high evolvability of new enzyme functions [86] suggests that enzymes are in general mutationally robust for their native functions, while at the same time accepting mutations that enhance promiscuous functions. An apparently neutral mutation may therefore actually be adaptive. Even an apparently detrimental (destabilizing) mutation might promote a promiscuous function that is only beneficial under certain environmental conditions that the experimenter may not be aware of.

The theory of neutral networks is impacted by the inclusion of degenerate native-state structures in that the notion of “neutrality” is moderated. While the strictest definition of neutrality (no change in protein activity/stability whatsoever) is usually not realistically applicable, a weaker definition (neutral, if the overall native structure is conserved, but a small loss of stability is tolerated) can be reconciled with experimental data. One can also go one step further and define neutral networks as *fuzzy sets*, where set membership is a continuous (not a binary) function over the interval 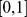 [87]. Degenerate native states could be easily incorporated into such a definition. Our biophysical model shows that, at least in theory, excited state conformations may contribute to promiscuous functions, and could therefore be included into the “fuzzy” neutral set of all sequences that have some non-zero probability of forming that conformation. The membership to a fuzzy sequence set could be provided by the fractional population of the conformation (Equation 1 in Methods). Neutrality depends on the strength of the selection pressures involved, so that membership to a fuzzy neutral set as defined above requires a certain threshold of minimum stability. In the same manner as a falling sea level will expose more habitable land mass, a reduced selection pressure will allow for a larger number of viable protein variants.

### Can evolvability be promoted by degenerate native states?

The intrinsic mutational robustness of neutral networks has been proposed to promote evolvability, i.e. the capacity to evolve towards new phenotypes [57], [88]. High robustness allows a population to accumulate many neutral variants within a neutral network. Some of these variants may be mutationally close to other phenotypes. We have shown that the inclusion of proteins with degenerate native states into neutral networks also enhances evolvability by providing more viable sequences between neutral networks. Compared to only proteins with non-degenerate native states, these additional sequences can access a substantial number of additional phenotypes. However, strong selection pressures would generally prevent evolution from utilizing degenerate native states, especially if only one of the native states is beneficial. The higher the native-state degeneracy, the lower the stability of a particular structure (see Figure 1b), and the lower the selection pressure would have to be for viability. If more than one native-state structure is beneficial, and if fitness effects are additive, a low stability may be compensated by providing multiple beneficial structures. Therefore, evolvability requires weak selection pressures in our model.

Draghi et al. [88] have found an analytical solution to the general problem of how robustness and evolvability are related. Their results are general enough to be applied to any system (biological or non-biological) that exhibits robustness. In particular, they have provided a biological example of RNA phenotypes. However, their study does not provide any information specific to proteins, because the necessary parameters cannot be measured easily. Proteins are fundamentally different from RNA: structure formation in proteins is largely determined by hydrophobic-polar interactions, which are largely absent in RNA. Consequently, proteins and RNA do not share similar genotype-phenotype relationships [89]. The results from our simple protein model are consistent with the general predictions by Draghi et al. that evolvability increases with robustness, given two conditions: first, robustness is relatively low (only 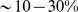 of mutations in sequences belonging to the same neutral network are neutral in our model; detailed data not shown); and second, only a small fraction of phenotype space can be accessed from each point in genotype space (true for our model, since the number of mutations per sequence is limited to 18, while phenotype space consists of 1475 stable structures [22]). One of the measures of evolvability that they use, and that we also have used here, is the number of mutationally accessible new phenotypes per genotype. An alternative measure is the time (e.g. number of generations) a population takes to adapt to a new beneficial phenotype. These two measures, however, capture different aspects of evolvability: one is the potential to quickly access many different phenotypes if the need arises (a concept followed by some experimentalists working on promiscuous enzymes [90]), while the other is adaptation to one specific phenotype that is under selection (a scenario we have investigated previously [32]). Here, we have followed the first approach of measuring evolvability, because we also impose the important additional requirement of conservation of the existing phenotype. With this restraint, the new beneficial phenotype is never fully reached by adaptation, especially since we refrain from a binary definition of neutral network membership (see previous section). Dual phenotypes (as exhibited by bi-stable proteins) have not been considered by Draghi et al. or any other theoretical study on neutral networks. By allowing dual phenotypes, which evidently also exist in nature, we allow an evolutionary compromise, whereas a binary definition of neutral networks completely prohibits adaptation as long as the need for conservation exists. In addition, our results also have consequences for the case of “unopposed” adaptation (without conservation), at least as far as modeling efforts are concerned: the true connectivity (evolvability) between neutral networks could be significantly underestimated, if proteins with degenerate native states are not considered. Both scenarios — a complete shift of selection pressures from one phenotype to another [32], [88] and adaptive conflict (present study) — are important fields of investigation since both are likely to exist in nature.

The true robustness and evolvability parameters of proteins remain largely unknown. It appears plausible, however, that proteins may have become the dominant type of biopolymer (as opposed to RNA, or other unknown biopolymers that might have existed during early stages of evolution), in part because they produce the right balance between robustness and evolvability that allows for fast adaptation.

### Future research on protein bi-stability and evolution

Bi-stability as a factor for protein evolution (as opposed to conformational changes that are part of the same protein function) is currently based on a few mostly artificial example cases, but has not been widely observed in natural settings. This may be caused, in part, by experimental limitations in protein structure determination, and possibly also by a lack of research focus. Conformational diversity, as a more general case of bi-stability, has only recently gained broader attention [11], [59], [91], but much of its potential for evolution remains unexplored. We propose that bi-stability is particularly beneficial in complex and quickly changing environments that are likely to create adaptive conflicts. One important example could be the evolutionary arms-race between hosts and parasites. Bacteria and viruses have limited genetic material for adaptation to act upon, therefore these organisms might benefit from bi-stable and thus bi-functional proteins. Further studies in this direction will be instructive.

## Methods

### Simple biophysical protein chain model

Our model folds polymers of length 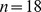 that are configured on a two-dimensional square lattice. The model sequences have a binary residue alphabet (H for hydrophobic, P for polar). This simplicity makes it possible to enumerate all possible structures, or conformations (self-avoiding walks on the lattice) for all 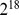 HP sequences. The energy function only includes one type of favorable energy, which is assigned for each hydrophobic intra-chain contact in any of the structures. Despite the simplicity in its construction, short-chain two-dimensional HP models have been shown to capture the essential physics of the sequence to native structure mapping of real proteins [29], [52]. The simplicity of the HP model allows for exact computation of the partition function — which takes full account of the energies of all structures, and thus permits an exact determination of the fractional population 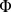 of each structure, which we use here as a stability measure. Specifically, 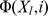 gives the probability of a protein with sequence 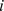 to fold into (adopt) structure 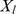:
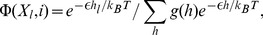
(1)where 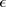 is the energy per hydrophobic-hydrophobic (HH) contact, 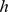 is the number of such contacts in a conformation (thus total energy 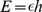), 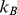 is the Boltzmann constant and 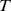 is absolute temperature. Conformation 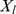 has 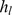 HH contacts. The summation in Equation 1 is over all possible 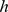 values in the entire conformational space 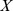, and 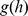 denotes the density of states of sequence 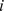 [23]. For any given HP sequence 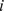, the native-state degeneracy 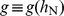 is the number of structures with the highest number of HH contacts, 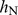. In the present study, 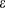 and 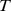 were chosen to provide conditions generally favorable to the folding of 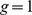 sequences. As in some of our earlier studies [23], we have used 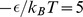 throughout the present work.

If the number of HH contacts in 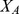 and 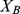 are denoted by 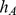 and 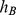, respectively, the difference 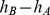 for a given sequence 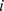 is a measure of stability difference for that sequence (as used in Figure 2) because 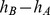 is directly related to the fractional populations 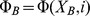 and 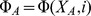, viz., it follows from Eq. 1 that
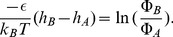
(2)The system of two adjacent neutral networks that we showed in Figures 2 and 4 as examples comprises one core neutral network (A; blue) with 48 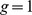 sequences or the corresponding extended neutral network that includes an additional 84 sequences with 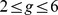, as well as another core network (B; red) with 20 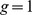 sequences or the corresponding extended neutral network that includes an additional 40 sequences with 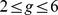. The Hamming distance between the two prototype sequences is 2, and the intra-chain contact difference between the native-state structures 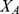 and 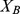 is also 2 (Figure S2).

### Calculation of fitness

In our model, the fitness of an HP sequence 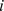 evolving under selection for two beneficial structures 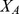 and 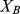 is given by
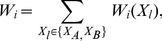
(3)where
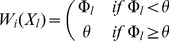
(4)and 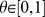 is an upper bound for the contribution of stability 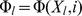 to fitness [81]. In all the computational results presented in this paper except those in Figure 5, the same 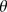 was assumed for 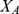 and 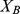 for simplicity, whereas two different upper bounds 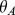 and 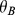 were used to gain a broader perspective in Figure 5. The upper bounds serve as a selection pressure because a low 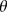 allows for destabilization of the protein, without fitness costs, whereas a high 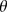 does not tolerate destabilization.

### Calculation of sequence populations at evolutionary steady state

Let 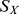 be the set that contains all sequences in the extended neutral networks of two structures 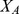 and 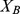. In our master-equation formulation of population dynamics [30], [81], the population of sequence 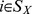 at time 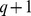 is a function of sequence populations at time 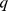:
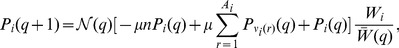
(5)where 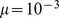 and 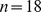 are, respectively, the mutation rate and sequence length chosen for the present study. 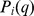 is the population of 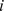 at time 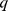, and 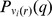 is the population of the 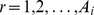 adjacent sequences of 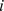, denoted here by 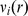, in the sequence network 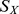, where two sequences are adjacent if and only if they can be converted to each other by a single mutation. The factor 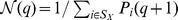 is introduced to keep the total population normalized to 1 to facilitate comparisons of 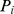 distributions at different time steps. The factor 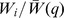 is a reproduction term that is determined by the relative fitness of sequence 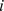, 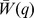 being the average fitness (weighted by population) of all 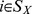 at time 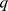.

Population dynamics were calculated from an initial state (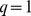) in which only the prototype sequence of one network (A) was populated (Figure S4). The steady state was reached by iterating Eq. 5 until the 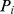 values remained essentially unchanged over many generations. For a given network topology, the steady state is independent of the initial state. In this regard, it should be noted that for some of the control calculations in Figure S3 only the initially populated network were populated at steady state because in those cases the two networks were disconnected by the artificial removal of bridge sequences in the control simulations.

### Monte Carlo simulations of finite populations

The above master-equation approach presupposes an effectively infinite population. To assess the effect of finite population on steady-state distributions, we have also conducted Monte Carlo simulations under the same general conditions with respect to selection pressure and mutation rate (Figure S4) [81]. Similar to the initial conditions in the master-equation formulation, every Monte Carlo simulation was initialized with a population consisting of 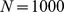 identical individuals each carrying the prototype sequence 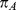. At each subsequent time step, a random number between 0 and 1 was drawn for each of the 18 monomers in each of the 1000 sequences. If the random number was less than 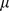, the monomer was mutated (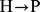 or 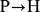, depending on whether the initial monomer was H or P), and fitness was then recalculated in accordance with Equation 3. Multiple mutations in one sequence can occur in one time step; but these events were very rare under the chosen value for 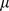. Evolution thus proceeded essentially in discrete steps of single point mutations. After all mutations were performed for a given time step, a new population was selected for the next time step by the following consideration: As in the master-equation formulation, the relative fitness of individual 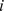 in the population with fitness 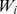 is 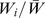, where 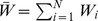. Let 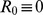 and 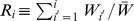 for 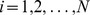 (thus 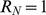). The 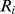
's resulting from this construction are the boundaries of 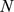 discrete bins in 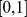 with widths equal to the 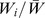 values. Now, to select an individual, a random number 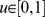 was drawn and individual 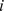 was selected if 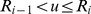. This procedure picks an individual by letting the random number fall into one of the 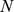 bins. By repeating this procedure 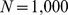 times, a new population of 1,000 individuals was selected. Because the same individual could be picked multiple times and some individuals might not be picked at all, fitter individuals would be statistically over-represented in the next generation, as they should.

### Neutral network layout

For illustrative purposes, the sequences belonging to the two adjacent neutral networks in Figure 2a were depicted as nodes placed by the Fruchterman-Reingold algorithm [92] that simulates physical spring forces between connected nodes. This algorithm serves to keep edge lengths as equal as possible, resulting in a network layout that roughly reflects the sequence connectivity relationships, i.e. sequences differing by many mutations are also farther apart in the two-dimensional node layout. Stability difference 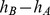 (see above) was then added as a third axis for the drawing in Figure 2a.

### Mutagenesis and energy calculations for the 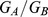 system

The NMR model 1 of 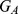 (PDB code 2FS1) and the X-ray structure of 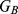 (PDB code 1PGA) were used as the wildtype structures in our analysis. The two wildtype sequences have a sequence identity of around 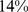. In addition to the wildtype pair, we considered also the sequence pairs in Refs. [27], [69] that are intermediate mutants between the two wildtypes and have pairwise sequence identity of 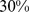, 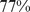, 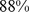, 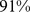, 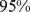, and 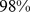. For any one of these sequences, only one — but not both — of the 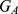 and 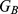 structures was experimentally inferred to be native [27], the other was a hypothetical excited-state structure. To estimate the stability difference between excited- and native-state structures, we modeled the free energy of every sequence in *both* the 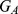 and 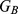 structures by “threading” each mutant sequence into a *modified* 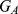 and a *modified* 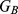 structure that were locally optimized for the given sequence. Two different methods, namely Rosetta and FoldX, were employed for this computation.

In the Rosetta approach (PyRosetta v2.0 implementation [93]), mutations were introduced using the “PackRotamersMover” routine to produce the sequence variants, then each of the two wildtype PDB structures embodying the mutant sequence were optimized using the FastRelax method, which is currently the best-performing free energy minimization method of Rosetta [71]. FastRelax was applied three times in a row to each wildtype PDB structure to ensure that the resulting structures were as optimized as possible and had comparable free energy scores. The same FastRelax procedure was also applied to the two wildtype sequences. Free energy scores were computed by the standard energy function of Rosetta with undamped Lennard-Jones repulsions (“hard rep”) [72].

In the FoldX approach, the mutagenesis engine (“BuildModel”) and the standard energy function of FoldX were used to generate and evaluate the sequence variants. For each sequence, the “Repair” function of FoldX was used to optimize the side-chain orientations. In contrast to the Rosetta approach that allows for movement of all atoms to achieve local optimization of the structure, FoldX (version 3.0) [70] only optimizes side-chain orientations but leaves the backbone unchanged, resulting in less structural optimization (from the PDB wildtype) for any given sequence. A comparison of the performance of Rosetta and FoldX in our analysis of the 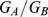 system is provided in Figure 3c.

### Intra-chain hydrophobic contacts in PDB structures

To determine the hydrophobic contact density 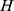 for a given all-atom protein structure (Figure 3d), the number of C-atom pairs from different amino acid residues and the total number of inter-residue atomic contacts were counted. An atomic contact is defined by an inter-atomic distance of less than 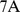. Computation of contacts was performed using the P3D Python module [94]. Among several possible choices of threshold separation, we found that a threshold separation of 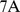 in the definition of 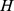 produced the best illustration of the native-structure switch between 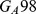 and 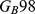 (Figure 3d). As in the lattice HP protein model [73], only contacts between residue pairs that are at least 3 positions apart along the chain sequence were counted in the 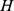 measure. Conceptually, the difference in hydrophobic contact density 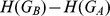 plotted in Figure 3d for the all-atom protein structures corresponds roughly to 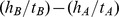, where 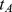 and 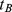 are, respectively, the total number of contacts of structures 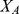 and 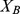 in our biophysical protein chain model. We note that 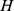 is a simple measure of hydrophobic contact density that does not rely on a hydrophobicity scale (e.g., that of Kyte-Doolittle [95]). It takes contributions from the carbon atoms in hydrophobic as well as non-hydrophobic residues. For instance, in the present application to the 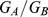 system, 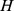 contains contribution from the C-atoms in the polar residue lysine in the core of both 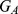 and 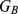 [27].

### Protein conformational diversity dataset

All 7989 redundant protein structure clusters were obtained from the protein conformational database PCDB (version 2, August 2011) [59]. Each entry in PCDB contains a cluster of CATH [64] domain structures that correspond to the same sequence. The largest conformational difference (max PCD) between two structures of the same cluster was determined, using the RMSD values (in 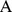) that were already included in PCDB (obtained using MAMMOTH [59], [96]). Stability value of each structure in a pair with max PCD was calculated with Rosetta [93] by the standard energy function (see above). If a structure had unfavorable energy (), the FastRelax method (see above) was applied until a favorable energy (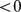) was reached. Potential bridge proteins were identified by the criteria described above in Results. The set of proteins we thus obtained is listed in Dataset S1 with the cause(s) of conformational diversity provided by PCDB.

## Supporting Information

Dataset S1 Excel table containing accession numbers of potential bridge proteins in the PCDB database.
(XLS)

Figure S1
**Bi-stable bridges are mutationally closer to a prototype sequence than non-bridge bi-stable proteins are.** The general plotting convention of the box plots are the same as that provided in the caption for Figure 1b in the main text. The median Hamming distance to the nearest prototype sequence for the non-bridge sequences (grey box) and for the bridge sequences (magenta box) are, respectively, 2 and 1. (In both of these cases, the median coincides with the first quartile, i.e., the lower bound of the box). The detailed procedure for constructing this figure is as follows. First, for each sequence with 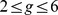, the prototype sequences of all of the neutral networks for the multiple native-state structures of that sequence were identified. In the second step, the shortest Hamming distance among the Hamming distances between the given sequence and each of the prototype sequences was determined. This procedure was repeated for all the bridge and non-bridge sequences to produce the statistics shown. By definition, bridge sequences are connected by single point mutations to at least two of the neutral networks of their native structures; whereas non-bridge sequences do not have this property. The statistics for 3987 bridge sequences and 19129 non-bridge sequences in our biophysical protein chain model are summarized in the present figure. Non-bridge sequences for which none of their multiple native structures form neutral networks (i.e., no sequence with 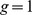 exists that folds into those structures) were excluded in this analysis because in that case there are no prototype sequences for the non-bridge sequence's multiple native structures.(TIFF)

Figure S2
**Example model protein structures.** The prototype sequences 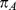 (**a**) and 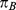 (**b**) of neutral networks A and B used in the main text are shown in their native structures 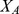 and 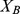. Black beads are hydrophobic residues, white beads are polar residues. Intra-chain hydrophobic contacts are indicated by orange dashed connections between beads. The sequences differ at three positions (labeled 1,2, and 3), and the structures differ by 2 intra-chain contacts (arrows).(TIFF)

Figure S3
**Steady-state distributions under adaptive conflict for several neutral network pairs.** Same as Figure 4 in the main text but generalized to other neutral network pairs. The largest six neutral networks (left hand sides; blue areas), each with a neighboring network (right hand sides; red areas), were chosen for evolutionary simulations (**a–f**). The overlapping region of the two networks is shown in magenta. Figure 4 from the main text is reproduced in panel **a** to facilitate comparison. Steady state sequence populations (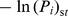 values) are plotted against Hamming distances of each sequence to the bridge protein with maximum stability. Negative distances indicate sequences from the initially populated (blue) network. The removal of bridges (grey symbols) disconnects neighboring networks in some cases, leaving only the left-hand network populated.(TIFF)

Figure S4
**Evolutionary dynamics under adaptive conflict leads toward bi-stable proteins.** Simulations were initialized with a population that was homogeneous for the prototype of network A (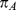), assuming previous strong conservation for only structure 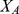. At the commencement of the dynamics simulation (first time step), a second selection pressure (for 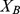) was introduced, thus creating an adaptive conflict. Selection pressures were intermediate for both structures (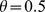) for this set of simulations. This condition allowed bi-stable proteins to evolve. Results from two different simulation approaches are presented. Master-equation (ME; solid curves) simulations are deterministic and assume an effective infinite population size (solid lines). We used this approach to compute the steady-state genotype distribution in Figure 4 in the main text. For comparison, simulations under analogous conditions were also performed using a stochastic Monte Carlo (MC; dashed curves) approach, where 100 finite populations of 1000 individuals each were simulated independently. For the ME simulations, genotype frequencies are given as fractions of 1 (left vertical axis). For the MC simulations, genotype frequencies are given as average numbers of individuals over 100 independent runs (right vertical scale). As in the ME simulations, the mutation rate in the MC simulations was 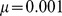 per monomer per time step. Time-dependent frequencies (time plotted along horizontal axis, in logarithmic scale) are shown in **a** for the following gene (sequence) categories: prototype 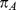 (blue diamond; dark blue curve), all 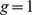 sequences from network A (blue circle; light blue curves), and the most stable bridge 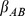 (magenta square; magenta curves). Panel **b** provides results from the control simulations in which bridge proteins were artificially removed from the neutral networks. Thus, 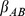 as well as other bridges cannot be populated. Instead, the prototype of network B (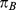; red diamond; darker red curves), and other 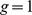 sequences from B (red circles; lighter red curves) become populated in this control case.(TIFF)
