# Supplementary figures and images for: Evolutionary Dynamics on Protein Bi-stability Landscapes can Potentially Resolve Adaptive Conflicts
Source: PLoS Comput Biol. 2012 Sep 13;8(9):e1002659. doi: 10.1371/journal.pcbi.1002659 (PMC3441461; doi:10.1371/journal.pcbi.1002659)

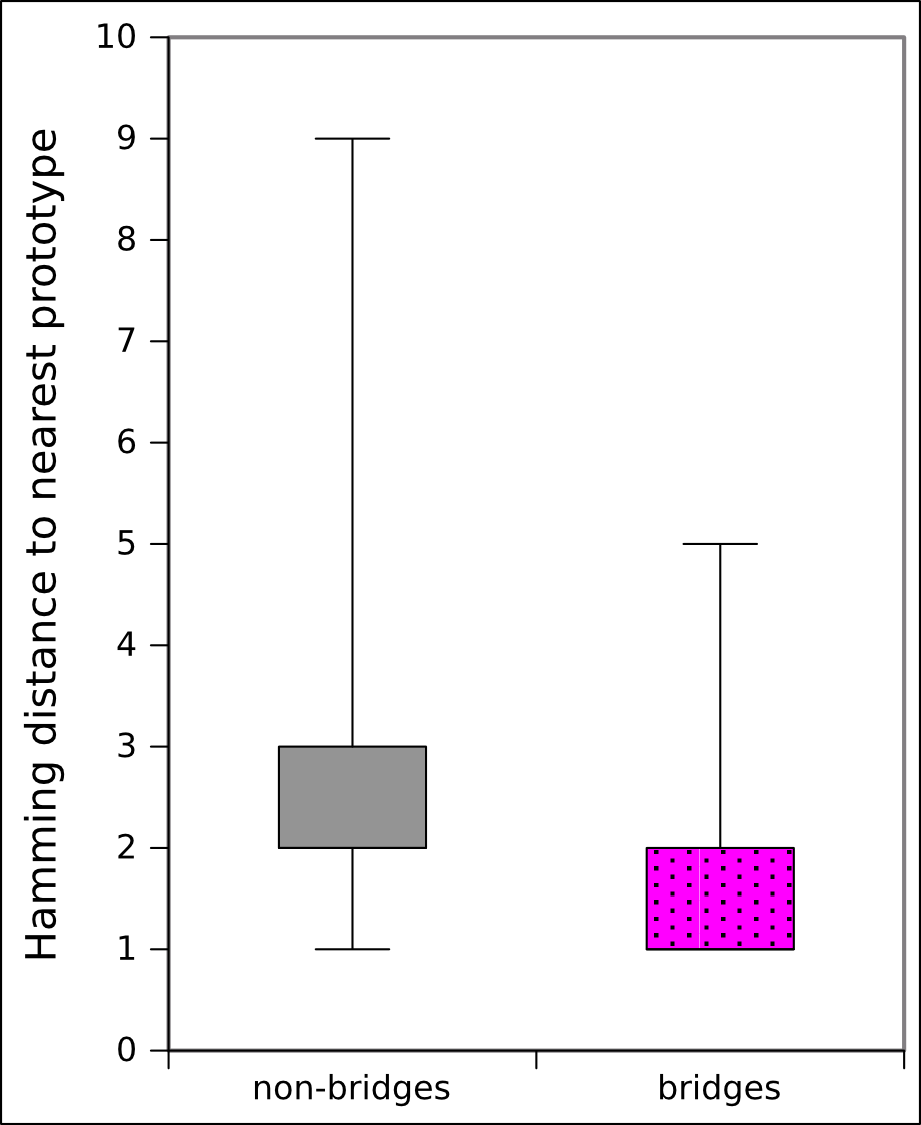

Supplement: Figure S1 — Bi-stable bridges are mutationally closer to a prototype sequence than non-bridge bi-stable proteins are. The general plotting convention of the box plots are the same as that provided in the caption for Figure 1b in the main text. The median Hamming distance to the nearest prototype sequence for the non-bridge sequences (grey box) and for the bridge sequences (magenta box) are, respectively, 2 and 1. (In both of these cases, the median coincides with the first quartile, i.e., the lower bound of the box). The detailed procedure for constructing this figure is as follows. First, for each sequence with , the prototype sequences of all of the neutral networks for the multiple native-state structures of that sequence were identified. In the second step, the shortest Hamming distance among the Hamming distances between the given sequence and each of the prototype sequences was determined. This procedure was repeated for all the bridge and non-bridge sequences to produce the statistics shown. By definition, bridge sequences are connected by single point mutations to at least two of the neutral networks of their native structures; whereas non-bridge sequences do not have this property. The statistics for 3987 bridge sequences and 19129 non-bridge sequences in our biophysical protein chain model are summarized in the present figure. Non-bridge sequences for which none of their multiple native structures form neutral networks (i.e., no sequence with exists that folds into those structures) were excluded in this analysis because in that case there are no prototype sequences for the non-bridge sequence's multiple native structures. (TIFF) [file pcbi.1002659.s002.tiff]

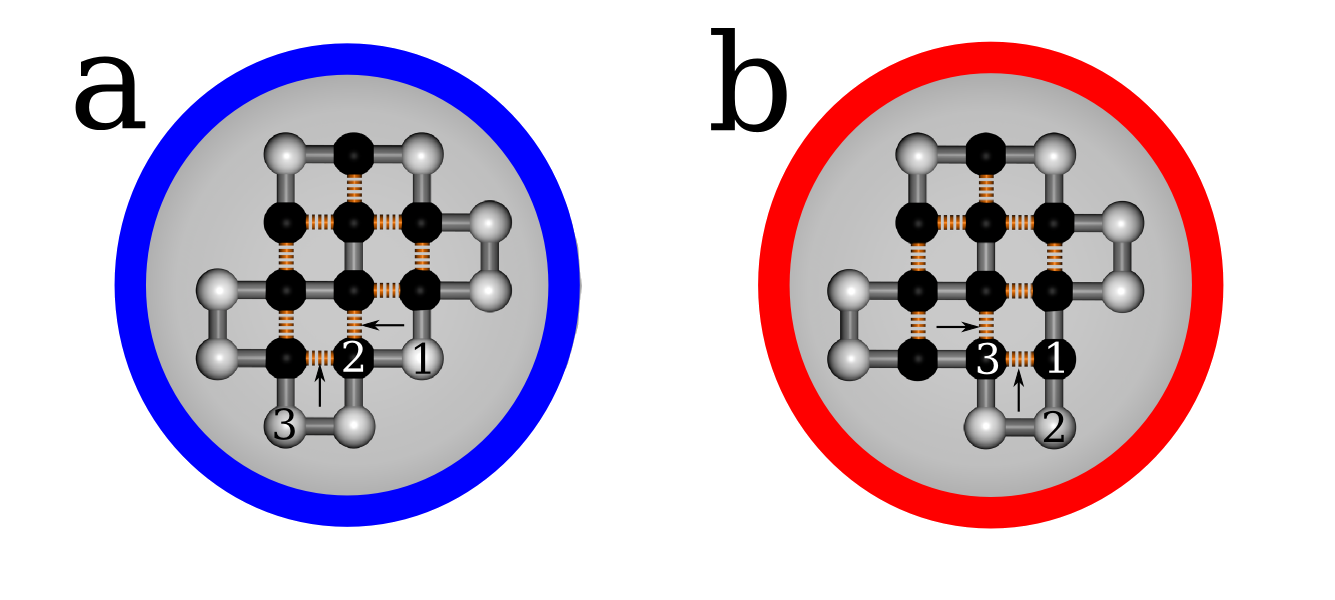

Supplement: Figure S2 — Example model protein structures. The prototype sequences (a) and (b) of neutral networks A and B used in the main text are shown in their native structures and . Black beads are hydrophobic residues, white beads are polar residues. Intra-chain hydrophobic contacts are indicated by orange dashed connections between beads. The sequences differ at three positions (labeled 1,2, and 3), and the structures differ by 2 intra-chain contacts (arrows). (TIFF) [file pcbi.1002659.s003.tiff]

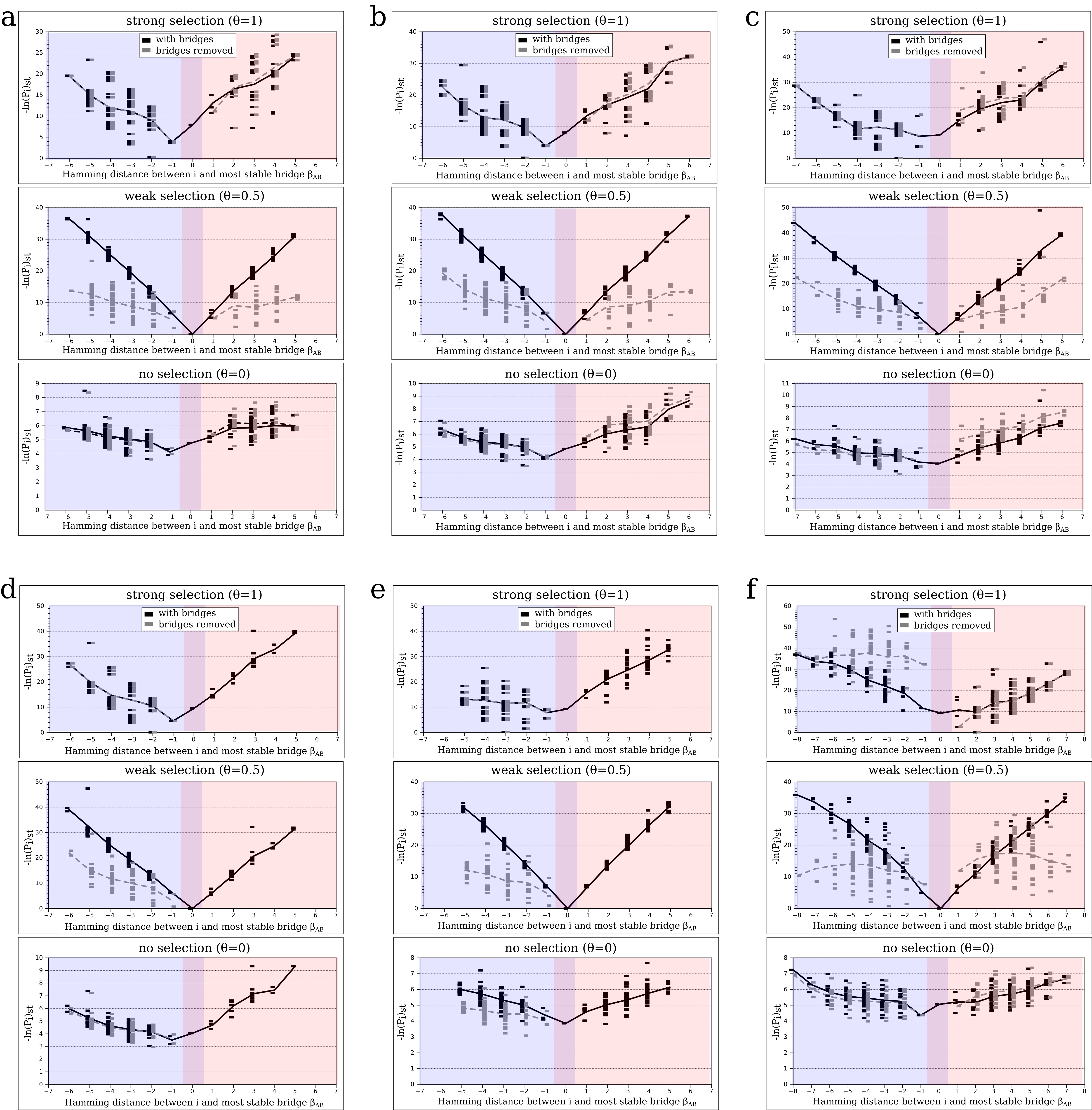

Supplement: Figure S3 — Steady-state distributions under adaptive conflict for several neutral network pairs. Same as Figure 4 in the main text but generalized to other neutral network pairs. The largest six neutral networks (left hand sides; blue areas), each with a neighboring network (right hand sides; red areas), were chosen for evolutionary simulations (a–f). The overlapping region of the two networks is shown in magenta. Figure 4 from the main text is reproduced in panel a to facilitate comparison. Steady state sequence populations ( values) are plotted against Hamming distances of each sequence to the bridge protein with maximum stability. Negative distances indicate sequences from the initially populated (blue) network. The removal of bridges (grey symbols) disconnects neighboring networks in some cases, leaving only the left-hand network populated. (TIFF) [file pcbi.1002659.s004.tiff]

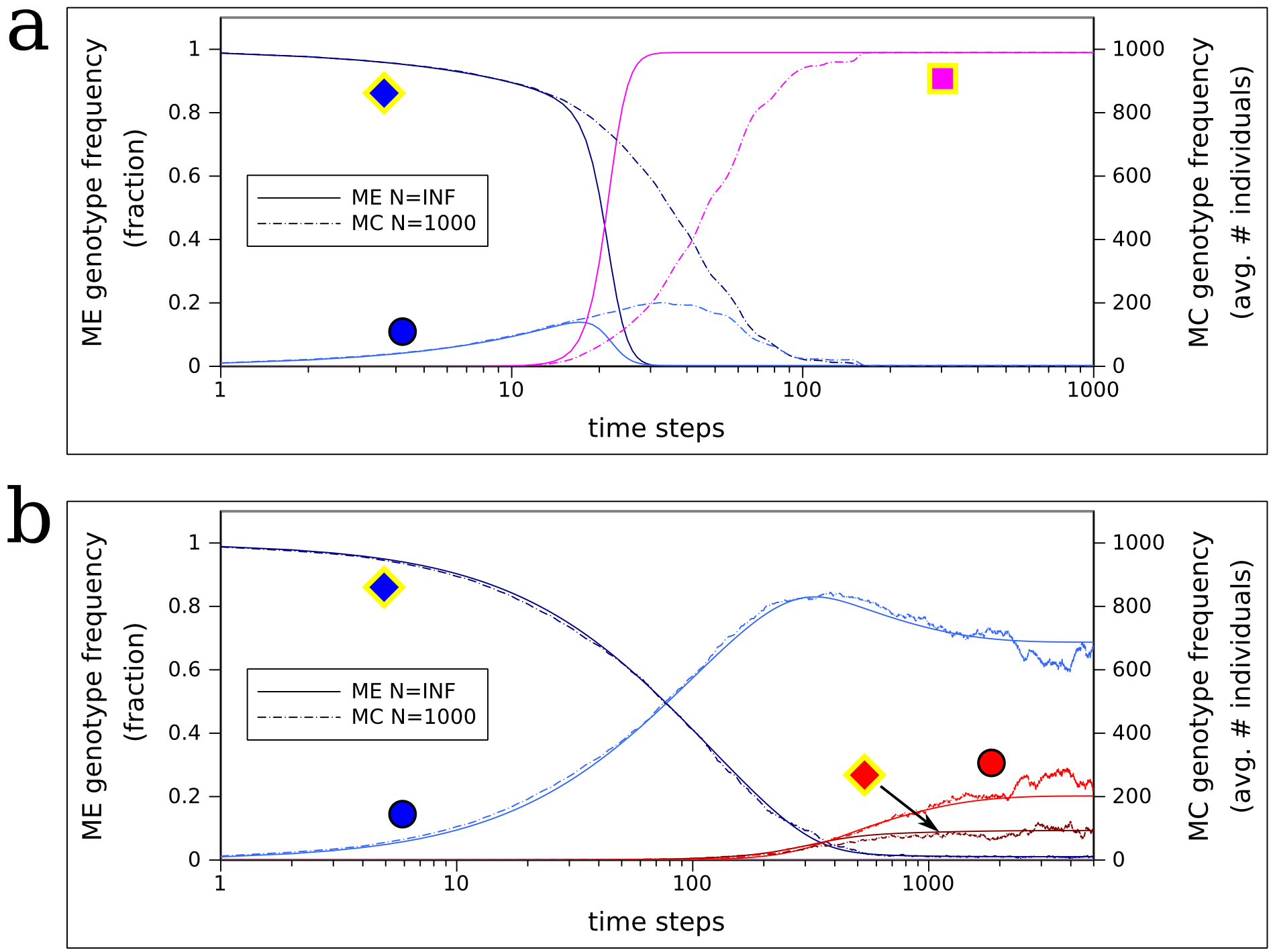

Supplement: Figure S4 — Evolutionary dynamics under adaptive conflict leads toward bi-stable proteins. Simulations were initialized with a population that was homogeneous for the prototype of network A (), assuming previous strong conservation for only structure . At the commencement of the dynamics simulation (first time step), a second selection pressure (for ) was introduced, thus creating an adaptive conflict. Selection pressures were intermediate for both structures () for this set of simulations. This condition allowed bi-stable proteins to evolve. Results from two different simulation approaches are presented. Master-equation (ME; solid curves) simulations are deterministic and assume an effective infinite population size (solid lines). We used this approach to compute the steady-state genotype distribution in Figure 4 in the main text. For comparison, simulations under analogous conditions were also performed using a stochastic Monte Carlo (MC; dashed curves) approach, where 100 finite populations of 1000 individuals each were simulated independently. For the ME simulations, genotype frequencies are given as fractions of 1 (left vertical axis). For the MC simulations, genotype frequencies are given as average numbers of individuals over 100 independent runs (right vertical scale). As in the ME simulations, the mutation rate in the MC simulations was per monomer per time step. Time-dependent frequencies (time plotted along horizontal axis, in logarithmic scale) are shown in a for the following gene (sequence) categories: prototype (blue diamond; dark blue curve), all sequences from network A (blue circle; light blue curves), and the most stable bridge (magenta square; magenta curves). Panel b provides results from the control simulations in which bridge proteins were artificially removed from the neutral networks. Thus, as well as other bridges cannot be populated. Instead, the prototype of network B (; red diamond; darker red curves), and other sequences from B (red circles; lighte [file pcbi.1002659.s005.tiff]
